# Transformation of the genital epithelial tract occurs early in California sea lion development

**DOI:** 10.1098/rsos.150419

**Published:** 2016-03-09

**Authors:** Cecilia Barragán-Vargas, Jorge Montano-Frías, Germán Ávila Rosales, Carlos R. Godínez-Reyes, Karina Acevedo-Whitehouse

**Affiliations:** 1 Unit for Basic and Applied Microbiology, School of Natural Sciences, Autonomous University of Queretaro, Avenida de las Ciencias S/N, Queretaro 76230, Mexico; 2 Department of Pathology, Instituto Mexicano del Seguro Social, Queretaro 76000, Mexico; 3 Cabo Pulmo National Park, Comisión Nacional de Áreas Naturales Protegidas, SEMARNAT, La Ribera, BCS, Mexico; 4 The Marine Mammal Center, 2000 Bunker Road, Sausalito, CA 94965, USA; 5 Sea Lion Cancer Consortium. http://www.smru.st-andrews.ac.uk/slicc

**Keywords:** California sea lion, DRB expression, MHC, otarine type I gammaherpesvirus, transformation, urogenital carcinoma

## Abstract

An unusually high prevalence of metastatic urogenital carcinoma has been observed in free-ranging California sea lions stranded off the coast of California in the past two decades. No cases have been reported for sea lions in the relatively unpolluted Gulf of California. We investigated occurrence of genital epithelial transformation in 60 sea lions (*n*=57 pups and 3 adult females) from the Gulf of California and examined whether infection by a viral pathogen previously found to be associated with urogenital carcinoma accounted for such alterations. We also explored the contribution of MHC class II gene expression on transformation. Cellular alterations, such as squamous cell atypia (ASC), atypical squamous cells of undetermined significance (ASCUS) and low-grade squamous intraepithelial lesions were observed in 42% of the pups and in 67% of the adult females. Normal genital epithelium was more common in male than female pups. ASC was five times more likely to occur in older pups. Epithelial alterations were unrelated to infection by the potentially oncogenic otarine type I gammaherpesvirus (OtHV-1), but ASCUS was more common in pups with marked and severe inflammation. Expression of MHC class II DRB loci (*Zaca DRB-D*) by peripheral antigen-presenting leucocytes showed a slightly ‘protective’ effect for ASC. We propose that transformation of the California sea lion genital epithelium is relatively common in young animals, increases with age and is probably the result of infection by an unidentified pathogen. Expression of a specific MHC class II gene, suggestive of presentation of specific antigenic peptides to immune effectors, appears to lower the risk of transformation. Our study provides the first evidence that epithelial transformation of the California sea lion genital tract is relatively common, even from an early age, and raises questions regarding differences in sea lion cancer-detection and -repair success between geographical regions.

## 1. Background

Cancer occurs in many vertebrates, is most common in mammals and is observed with a high incidence in only a few wild species that thus offer spontaneous models for the study of carcinogenesis [1]. Currently, cancer is a major health problem worldwide and is considered an important cause of disease and mortality in humans and companion animals. In most cases, cancer has a complex and multifactorial aetiology, with genetics believed to play an important role [2]. Cases of cancer in wild mammals have mostly been rare, typically reported as single stochastic occurrences (see [3,4] for reviews). The recent emergence of contagious and highly aggressive facial tumours in the endangered Tasmanian devil (reviewed recently by Belov [5]) has drawn international attention to wildlife cancer and has emphasized the importance of understanding risk factors and carcinogenesis [1]. While a number of studies have investigated epidemiological associations of cancer with specific factors such as persistent pollutants (e.g. [6,7]), mechanistic associations are yet to be determined [3,4].

Carcinomas of urogenital origin (UGC) were first reported in the 1980s [8]. However, since the mid-1990s, cases have been detected increasingly in adult California sea lions (*Zalophus californianus*) stranded along the central California coast and are now diagnosed in 26% of adult animals examined at necropsy [1]. Typically, infiltrative tumours and intraepithelial neoplasia are found in the cervix and vagina of UGC-affected females, and penis and prepuce of affected males [9,10]. Histologically, sea lion UGC shares many traits with cervical neoplasia in humans, including the anatomical pattern of metastases [11].

As happens with most types of cancer, there are a number of intrinsic factors that appear to be associated with occurrence of sea lion UGC. The causal or mechanistic link for such associations remains unknown. However, interesting correlations have been reported. For instance, otarine type I gammaherpesvirus (OtHV-1) [10,12], and beta-haemolytic streptococci [13] have been linked to cases of UGC in the California sea lion and other pinniped species [14]. While the role of beta-haemolytic streptococci in carcinoma aetiology has been questioned [1], detection of OtHV-1 in sea lion genital tumours is especially interesting, as the oncogenic role of gammaherpesviruses is well known for other animal species. For instance, in humans, the Epstein–Barr virus, a gammaherpesvirus closely related to OtHV-1 [15], induces genomic instability by altering expression of key genes, thus leading to cancer [16,17]. Several human papillomavirus (HPV) strains are also associated with the development of cervical cancer, although infection does not always lead to cancer [18]. It is as yet unknown whether OtHV-1 is indeed oncogenic for California sea lions, or whether its presence is unrelated to the pathology.

Genetic factors have also been reported to influence occurrence of sea lion UGC. For instance, lower levels of heterozygosity are more common in UGC-afflicted sea lions [19]: this finding is explained by a combination of inbreeding [19,20], and associative over-dominance in genes under selection (e.g. the heparanase 2 gene [21]). Also, the presence of a specific MHC class II DRB locus, named *Zaca DRB-A*, increased the risk of UGC nearly fourfold [22].

In contrast with the marked increase in cases of UGC in sea lions from the species’ northern breeding distribution, this condition has yet to be reported in sea lions from the Gulf of California, even though mortality and pathology surveys are conducted routinely [23]. Taking into account that the California sea lion provides an unusual opportunity to study spontaneous carcinogenesis [1], it is relevant to question whether the apparent absence of UGC in the Gulf of California might be due to differences in occurrence or prevalence of the previously identified risk factors. We know that levels of heterozygosity do not differ between these geographical regions [24] and there are no significant differences in the MHC class II DRB loci frequency [25]. However, a recent study found that sea lion blubber levels of polychlorinated biphenyls and organochlorine pesticides are two orders of magnitude lower in the Gulf of California than what has been reported for animals stranded in California [26–28], and high concentrations of these organic pollutants have been related to increased death risk due to UGC in adult California sea lions [28].

To date, surveys to determine the presence of potentially oncogenic OtHV-1 in sea lions from the Gulf of California have not been conducted, and neither has the possibility that precancerous transformation of the genital epithelium occurs in these animals been explored. Owing to the species’ migratory behaviour and natural history, we predicted that OtHV-1, which is most likely host-adapted and must harbour a long coevolutionary history with their host, will be present in sea lions from within this geographical area in similar levels to what has been recorded for other areas of the sea lions’ distribution. If OtHV-1 is detected, taking into account the absence of reports of UGC in animals from this area, these pathogens might have a lesser role as causal factors for sea lion UGC than has been proposed. Thus, in order to test our predictions, we conducted thin-layer cell smears of the genital tract to search for cellular phenotypes typically considered as precancerous or transformed cells in humans and mice [29–31], and performed qualitative and quantitative molecular assays to examine the occurrence of OtHV-1 in the genital swabs. We also tested the importance of constitutive and expressed MHC class II DRB diversity for (i) epithelial transformation and (ii) infection by the presumed oncogenic OtHV-1.

## 2. Material and methods

### 2.1 Sample collection

Fifty-seven California sea lion pups (*n*=24 male and 33 female) and three adult females were sampled in 2012 at Granito rookery (located on Granito Island; 29°33′43′′ N, 113°32′04′′ W), in the Midriff region of the Gulf of California during three sampling seasons: July (*n*=28 6–8 week old pups and 3 adult females), October (*n*=19; cohort of 18–20 week old pups) and January (*n*=10; cohort of 30–32 week old pups). None of the pups sampled at one season were resampled at a later season.

Sea lions were captured using hoop nets and were manually restrained. Genital epithelial swabs were collected using sterile cytobrushes. For male pups, the penis was manually exposed by pushing the prepuce back and the penile shaft was gently swiped longitudinally 8 to 10 times. For female pups, the cytobrush was carefully inserted into the cervix and swiped 8 to 10 times using circular movements. The adult females were sampled as indicated above, but a sterile vaginal speculum was used to reach the cervix. Three swabs were collected as described above from each pup; one was stored in a vial containing 96% ethanol while the other swabs were used to make cytology smears. Slides were chemically fixed on site by spraying with Cytofix^®^ fixative spray as per the manufacturer’s instructions (BD Biosciences, USA) and were protected from sunlight until staining. A 7 ml blood sample was collected from the caudal gluteal vein using a heparin-coated vacuum tube (Vacutainer^®^, BD Biosciences). Blood was homogenized and centrifuged immediately on site to separate the buffy coat, which was preserved with RNAlater (Qiagen, USA) and kept in a liquid nitrogen dry shipper until processing. A 3 mm^2^ skin sample was collected from the third digit of the right hind flipper and preserved in 96% ethanol.

### 2.2 Isolation and purification of nucleic acids

Genomic DNA was extracted from the ethanol-preserved genital swabs and skin samples using a proteinase K digestion followed by a phenol–chloroform protocol and isopropanol precipitation [32]. DNA was quantified and quality-assessed in a NanoDrop (Qiagen) spectrophotometer.

Total RNA was extracted from the buffy coat samples using Trizol reagent (Sigma, USA) to determine MHC Class II DRB (hereafter *Zaca DRB*) expression patterns of circulating antigen-presenting cells (APC). Fifteen micrograms of RNA were reverse-transcribed using Quantitect Reverse Transcription Kit (Qiagen). DNA contamination was avoided by the addition of DNAse I (2.5 U/reaction) during RNA extraction [32].

### 2.3 Detection of OtHV-1 DNA

We amplified a 510 bp fragment of the DNA polymerase (Dpol) gene of the type I otarine gamma herpesvirus in DNA extracted from the genital swabs (Dpol 697 5′-GCGGGAACGCAACTATATCCT and Dpol 65 5′-TCTTCGTCCAGTATCATTG) [33]. All 12.5 μl PCR reactions were performed using an ABI 3100 PCR system (Applied Biosystems, Inc.) and were conducted in duplicate. Cycling conditions were 95°C for 15 min, 40 cycles of 94°C for 40 s, 52°C for 30 s and 72°C for 40 s and a final extension step at 72°C for 10 min.

Amplified products were electrophoresed on a SYBR gold-stained 1% agarose gel, and visualized on a UV transilluminator. To ensure the amplified products were not the result of non-specific amplification, bands were gel excised, column purified (QiaQuick, Qiagen), cloned and bidirectionally sequenced for confirmation. Each sequence was visually inspected and compared with those reported in GenBank (http://www.ncbi.nlm.nih.gov/genbank/).

### 2.4 MHC typing and expression assays

Genomic DNA was examined with 10 sequence-specific primer pairs (SSP) [34] flanking the putative peptide-binding site of the California sea lion reported *Zaca DRB* genes A through J [22,25]. Buffy coat cDNA was analysed by SSP-PCR to determine the expression (presence/absence) of each of the 10 *Zaca DRB* genes. A housekeeping gene, glyceraldehyde-3-phosphate dehydrogenase (GAPDH), was used as an internal control for amplification. SSP-PCR was performed on an ABI 3100 PCR system (Applied Biosystems) and each reaction was conducted in duplicate under the following cycling conditions: initial denaturation at 94°C for 3 min, followed by 35 cycles of 94°C for 30 s, 58°C for 30 s and 72°C for 45 s, with a final extension step of 72°C for 10 min. Amplified products were visualized on a 1.5% agarose gel stained with ethidium bromide. Constitutive and expressed *Zaca DRB* multiplicity was determined as the number of genes detected in the skin DNA and in the buffy coat cDNA, respectively. These analyses were only performed on the 57 pups sampled.

### 2.5 Cytology

Epithelial slides were stained using a Papanicolaou protocol (PAP). This protocol allows the staining of nuclear chromatin, differential cytoplasmic counterstaining and cytoplasmic transparency [35]. The PAP stain was modified slightly to increase resolution. Specifically, 100 μl of potassium permanganate (KMnO_4_) were added to 90 ml of Harris haematoxylin stain, 20 μl of hydrochloric acid (HCl) were added to 60 ml of OG6 stain and 100 μl of acetic acid (C_2_H_4_O_2_) were added to 60 ml of EA50 eosin prior to staining the slides. Incubation, washing and decolourization times were run according to published protocols [35]. A 3′′×1′′ grid of 1 mm^2^ squares was placed beneath each slide to calculate the total smear surface under the stereoscope microscope. The total number of cells in the genital smears and the differential cell counts were calculated and standardized to cells per mm^2^. Cellular phenotypes taken into account were cells with a large, well-demarcated, clear perinuclear zone surrounded by a dense peripheral cytoplasmic rim [36] (hereafter, koilocytes); binucleated cells; cells with irregular chromatin and prominent nucleoli (hereafter, reactive cells) and atypical metaplasia. These three cellular phenotypes are typically considered markers of preneoplastic transformation in human genital smears [37]. We also counted lymphocytes, neutrophils and intranuclear inclusion bodies.

Each sea lion was evaluated for the presence of inflammation and dysplasia. Inflammation was categorized as none, mild, moderate, marked and severe, based on the number of inflammatory cells present. Each individual was placed in one diagnostic category as follows: negative for intraepithelial lesion of malignancy (negative), squamous cell atypia (ASC), atypical squamous cells of undetermined significance (ASCUS), low-grade squamous intraepithelial lesions (LSIL) and high-grade squamous intraepithelial lesions (HSIL), based on the 2001 Bethesda System Terminology [38]. Briefly, according to this cytological terminology, ASCUS includes cells for which a reliable interpretation of squamous intraepithelial lesions cannot be made although they contain features that are more marked than merely reactive changes [39] and its diagnosis should not be ignored as it often progresses to squamous intraepithelial lesions or malignancy [38]. LSIL includes mild dysplasia and cervical intraepithelial neoplasia grade I [40]. ASC refers to the presence of atypical cells that do not tend to transform to a malignant state and could be caused by infection by various pathogens, such as *Trichomonas vaginalis*, *Mycoplasma genitalium*, *Chlamydia trachomatis*, *Candida* and *Streptococcus agalactiae* [41–43].

### 2.6 Statistical analyses

For each diagnostic category we investigated the predictive value of *Zaca DRB* multiplicity [34], expressed *Zaca DRB* diversity, and the presence/absence of specific *Zaca DRB* expressed genes. For this, we built independent logistic regression models. The effect of explanatory categorical values was examined by the use of contingency tables.

The relationship between the presence of specific *Zaca DRB* genes and the number of each of the transformed cell types was examined by constructing a series of independent generalized linear models, which included sex and age (6–8 weeks, 18–20 weeks, 30–32 weeks, adult) as explanatory covariates. Before analyses were conducted, the distribution of each explanatory variable was assessed visually and deviations from normality were assessed by a Shapiro–Wilk test. The distributions of the transformed cell phenotypes (koilocytes, reactive cells, binucleated cells and atypical metaplastic cells) violated the assumptions for normality and showed a negative binomial distribution, and their values were subsequently log transformed. Heteroscedasticity, residual distribution and disproportionate influence of outliers were examined for each model [44]. The goodness of fit of each model was evaluated by comparison of the Akaike information criterion. All statistical analyses were performed in R v. 3.1.2 (Vienna, Austria).

## 3. Results

OtHV-1 was detected in the genital epithelial samples of 3.5% (*n*=2) of the pups and in 33.3% (*n*=1) of the adult females. The Dpol sequences generated from the OtHV-1 PCR products shared high nucleotide similarity indexes with sequences previously reported (California sea lion herpesvirus accession number AF236050, 91% identity, *E*-value e^−59^).

The genital epithelium of 57.89% of the pups sampled was classified as negative for intraepithelial malignancies. The genital epithelium of 21% of the pups was classified as ASC, 15.79% as LSIL and 5.26% as ASCUS. The genital epithelia of two of the three adult females sampled (66.67%) were categorized as LSIL. None of the 60 samples showed evidence of HSIL (see the electronic supplementary material for photomicrographs of the lesions). Thirty-three pups (57.89%) and three females (100%) had at least one of the following transformed cell types: koilocytes, binucleated cells and reactive cells, with varying numbers of each (table 1). Of these, the most prevalent in pups were reactive cells (35% prevalence; mean=1.24±4.29/100 mm^2^) followed by binucleated cells (33%; mean=0.11±0.11/100 mm^2^) and koilocytes (14%; mean=0.35±1.26/100 mm^2^), while adult females had higher numbers of koilocytes (mean=17.57±27.55/100 mm^2^). Intranuclear inclusion bodies were present only in two of the sampled sea lion pups (mean=0.01±0.05/100 mm^2^) and in one of the adult females (0.12/100 mm^2^). None of these individuals were positive for OtHV-1. Severity of inflammation increased with age (table 1).

**Table 1.** The prevalence of California sea lion genital epithelia that were negative for intraepithelial lesions of malignancy, and those that had mild to severe inflammation (infl.), squamous cell atypia (ASC), low-grade intraepithelial lesions (LSIL) and atypical squamous cells of undetermined significance (ASCUS). The median and range of normal epithelial cells, abnormal cellular phenotypes and immune cells that were quantified in the smear are shown for each age and sex class (referred to as cells/100 mm^2^).

	6–8 weeks old		18–20 weeks old		30–32 weeks old		
	M (*n*=8)	F (*n*=20)	M (*n*=10)	F (*n*=9)	M (*n*=6)	F (*n*=4)	adult females (*n*=3)
negative	85.71%	70%	62.5%	88.89%	16.67%	25%	—
mild infl.	14.29%	20%	12.5%	0%	50%	0%	33.33%
moderate infl.	0%	5%	0%	0%	0%	25%	66.67%
marked infl.	0%	0%	25%	0%	16.67%	50%	0%
severe infl.	0%	5%	—	11.11%	16.67%	0%	0%
ASC	0%	10%	50%	33.33%	33.33%	25%	0%
LSIL	16.66%	25%	0%	0%	0%	50%	66.67%
ASCUS	0%	0%	0%	22.22%	0%	25%	0%
normal cells	43.1	5.1	7.3	0.7	0	0.8	0.6
	(2–56.9)	(0.9–19.6)	(0–41.7)	(0.1–11.8)	(0–11)	(0–1.7)	(0–1.5)
koilocytes	0	0	0	0	0	0	3.4
	(0–0.1)	(0–5.1)	—	—	(0–3)	(0–7.1)	(0–49)
binucleated	0	0	0	0	0	0.1	0.1
	(0–0.5)	(0–0.4)	—	(0–0.1)	(0–2)	(0–0.5)	(0–0.6)
reactive	0	0	0	0	0	0	0
	(0–1.2)	(0–24)	(0–0.2)	(0–17.9)	(0–6)	—	—
atypical metaplastic	5.3	0	0	0	0	22.4	0
	(0–6.8)	—	—	—	—	(1.2–27.3)	—
intranuclear inclusions	0	0	0	0	0	0	0
	(0–0.3)	—	—	—	—	(0–0.2)	(0–0.2)
lymphocytes	0	1.1	0.9	3	3.5	8.5	3.9
	(0–0.1)	(0–27.6)	(0–5.2)	(0–16.6)	(0–10.6)	(0–23.6)	(2.7–5.0)
neutrophils	0	6.8	8.9	3.3	43.9	5.7	0.4
	(0–5.1)	(0.1–76.9)	(0–7.94)	(0.1–67.5)	(0.4–95.8)	(0.5–16.6)	(0–1.1)

**Figure 1.**
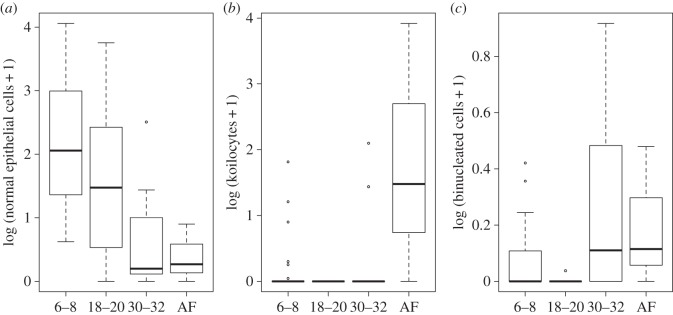
Age-related variation in the genital epithelium of California sea lions. (*a*) The number of normal epithelial cells decreased with age (*R*^2^=0.13; *F*_3,59_= 7.15, *p*=3×10^−4^); (*b*) koilocytes increased with age (*F*_3,59_=8.97, *p*=6×10^−5^), (*c*) binucleated cells were more abundant in older pups, and decreased again in adult females (AF) (*F*_3,59_=6.46, *p*=7×10^−4^). The figure shows the median (thick line), first and third quartile (box) and 95% CI of median (whiskers). The *x*-axis shows the sampled animals’ age in weeks (pups).

Some of the cellular phenotypes varied with age (figure 1; table 1). Specifically, the number of normal epithelial cells decreased with age (*F*_3,59_=7.15, *p*=3×10^−4^, figure 1*a*), while koilocytes increased with age (*F*_3,59_=8.97, *p*=6×10^−5^, figure 1*b*). Binucleated cells increased when pups were older, and decreased again in adult females (*F*_3,59_=6.46, *p*=7×10^−4^, figure 1*c*). The occurrence of moderate and marked inflammation (infiltration of lymphocytes and neutrophils) increased with age (moderate inflammation: Adj *R*^2^=0.08, *F*_1,58_=6.12, *p*=0.01; and marked inflammation: Adj *R*^2^=9.06, *F*_1,58_=4.59, *p*=0.03).

**Figure 2.**
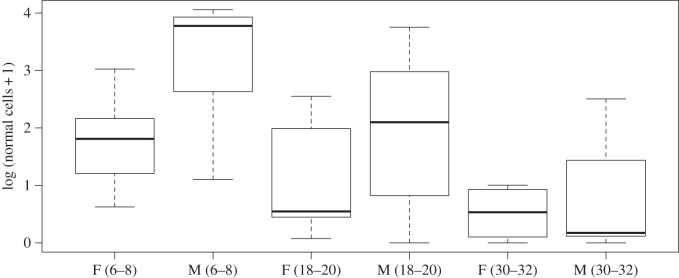
Sex-related variation in the number of normal epithelial cells of pups. Male pups had significantly higher numbers of normal epithelial cells at 6–8 weeks of age and at 18–20 weeks of age. The figure shows the median (thick line), first and third quartile (box) and 95% confidence interval of median (whiskers). The *x*-axis shows the sampled animals’ age in weeks and sex (F, female; M, male).

There were no intersex differences in the number of transformed cell types of pups for each age class (*p*>0.05 for each cell type); however, the number of normal epithelial cells tended to be higher in male pups (*F*_1,56_=6.83, *p*=0.01). When examining each pup age class separately, males had more normal epithelial cells than females at 6–8 weeks of age and at 18–20 weeks of age (figure 2).

Infection by OtHV-1 was not associated with any of the cell types or diagnostic category (*p*>0.05 for all models), nor was it related to the number of lymphocytes and neutrophils found in the cervical smear. Atypical squamous transformation (ASCUS) was more common in animals whose genital epithelium was marked and severely inflamed (*χ*^2^=25.74, *n*=60; *p*=3.6×10^−5^), and this pattern held when excluding adult females from the analysis (*χ*^2^=27.22, *n*=57; *p*=1.8×10^−5^). When examining this relationship in terms of the number of lymphocytes and neutrophils, sea lions with ASCUS tended to have more lymphocytes (*z*=2.21, *p*=0.027) but not more neutrophils than normal individuals.

Pups’ constitutive *Zaca DRB* multiplicity ranged from 6 to 10 genes (mean=8.81). Diversity of APC-expressed *Zaca DRB* genes ranged from 3 to 10 loci (mean=5.77). Neither constitutive *Zaca DRB* gene multiplicity nor expressed *Zaca DRB* gene multiplicity explained occurrence of LSIL, ASCUS and ASC ( *p*>0.05 for all models).

The presence or absence of specific *Zaca DRB* genes did not influence any of the cellular phenotypes or diagnostic categories (*p*>0.05 for all models). Similarly, individual expression of *Zaca DRB* genes did not appear to play a significant role in the occurrence of any of the abnormal cytological diagnostic categories, nor did it influence the number of abnormal cell types except for *Zaca DRB-D*, which showed protective effects. Specifically, pups that expressed *Zaca DRB-D* had a slightly lower risk of having ASC (*z*=−2.48, *p*=0.01). The number of infiltrated neutrophils and lymphocytes was not influenced by the expression of any of the 10 *Zaca DRB* genes (*p*>0.05 for all models).

## 4. Discussion

The high prevalence of California sea lion urogenital carcinoma detected in animals stranded along the central California coast has raised concerns about sea lion health and welfare and led to interesting questions about the pathogenesis and risk factors associated with this disease [1]. One of the most intriguing aspects of urogenital carcinoma could well be why it does not appear to occur in California sea lions in other areas of their distribution. We have now shown that transformation of the genital epithelium occurs in sea lions from other geographical regions and is detectable even at early life stages.

To date, our knowledge on the prevalence of urogenital carcinoma in sea lion populations has depended on having access to post-mortem examinations of stranded individuals [45,46]. This approach is extremely useful for obtaining detailed information on the morphology and aggressiveness of this type of cancer, but does not allow estimating the prevalence of the condition in natural populations. Our cytology-based approach sheds light on early stages of transformation, prior to the onset of cancer. Nearly half of the sampled sea lions revealed mild to moderate epithelial transformation of the genital tract, with categories that included LSIL and atypical squamous cells of undetermined significance. In humans, since establishing the diagnostic value of high-contrast staining of vaginal cells, these diagnostic categories have been considered the standard for detecting malignancies and early cancer of the cervical tract [47].

To date, urogenital carcinoma has been detected only in sub-adult and adult sea lions, and observed loss of oestrogen receptor (ER-*α*) expression has strengthened the suggestion that reproductive hormones and cofactors could play a role in malignant transformation and proliferation [45]. In this context, finding a moderate prevalence of LSIL in the sampled pups was unexpected, as they were far from being sexually mature. The only other study of sea lion genital cytology that we are aware of did not find evidence of dysplastic cells or cytologic traits of malignancy [13]. However, the previous study used Wright-Giemsa instead of PAP, a stain that is considered more sensitive for the detection of cellular malignancies [48,49], rendering comparisons of our findings difficult. We are unaware of studies on the juvenile genital cytology of other mammal species, and it is unlikely that PAP screening is routinely conducted in humans before sexual activity commences [50], so contrasting our findings of LSIL and ASCUS in young animals to what has been observed in other species is not yet feasible. However, it is worth noting that some animal species appear to be more prone to spontaneous oncogenesis than others [51,52], and cellular transformation has been known to occur at an early age [53]. It is possible that the California sea lion has a similar tendency to cellular transformation of the genital tract, which would help explain our results that suggest that epithelial transformation of the California sea lion tract can occur early in their life. If similar results to ours were to be found in animals from the breeding colonies near Northern California, the contrasting lack of cases of urogenital carcinoma in the Gulf of California would imply that, in animals from this region, transformed cells are successfully resolved or halted before the onset of cancer. Evidently, our study area is not the entire Gulf of California. However, Granito Island is considered one of the founder colonies of the area in terms of mitochondrial, microsatellite and MHC diversity [25,54], so it is likely to be a good colony-model for understanding biological processes related to California sea lions within the Gulf of California.

Measuring the concentration of organic contaminants in the sampled pups was beyond the scope of our study. However, one could hypothesize that if sea lion genital epithelium is indeed prone to spontaneous transformation, the reported differences in the concentrations of environmental organic contaminants (two orders of magnitude lower than those reported for sea lions in their more northern distribution [26–28]) could help explain the lack of urogenital cancer in the Gulf of California. In humans, genital epithelia with LSIL often does not progress to HSIL and cervical neoplasia when the cause of damage, in this case HPV, is kept under control by treatment or by a healthy immune system [55], and depending on the infecting HPV serotype [56]. For instance, a longitudinal study on more than 2000 Brazilian women showed that the majority of LSIL events detected by cytology regressed to a lower grade, and this was more common when papillomavirus infections were cleared [18]. Constant exposure to high levels of organic pollutants can certainly lead to development of cancer in dogs [57] and humans [58], and levels of organochlorines tend to be higher in sea lions diagnosed with urogenital carcinoma, compared with non-cancer animals [28].

In terms of the pathogens previously associated with urogenital carcinoma, the prevalence of OtHV-1 in the genital tract of the sampled animals was low. Prevalence in pups was similar to that found in the genital tract of pups sampled in US waters (between 1.5 and 12.5% [59]) and lower for adult females than reported for similarly aged sea lions from San Miguel Island, USA (19.6% [59]). In contrast with earlier studies, we failed to find evidence of an association between infection by OtHV-1 and any of the diagnostic cytology categories, or with the number of transformed cell types. These results suggest that OtHV-1 might not be a contributory factor towards initial transformation, and that this virus could play an important role later in the development of cancer, as has been observed in nasopharyngeal carcinoma patients [60]. Our findings also raise the possibility that other, as yet unidentified, pathogens could influence initial transformation of the genital epithelium. This possibility is supported by the fact that, in humans, two of the abnormal cell types here detected, koilocytes and binucleation, are typically associated with infection by papillomavirus, not herpesvirus [29]. To date there has been no evidence that papillomaviruses are associated with sea lion urogenital carcinoma [12], but their prevalence in apparently healthy genital tissue and their potential role as early initiators of transformation has not yet been explored.

An explanation of the relatively common ASC found in the genital epithelium of pups could be shifts in the local microbiome during neonatal development, as has been reported for humans [61] and mice [62], or alternatively, infections by other pathogens, such as *Candida, Chlamydia*, *Trichomonas* and *Mycoplasma* [41–43], some of which are common inhabitants of the genital tracts of otariid pinnipeds [13,63]. These possibilities need to be explored in the future.

It is interesting to note that the expression of *Zaca DRB-D* in circulating leucocytes had a slightly protective effect for developing ASC. Such an effect would be expected under a scenario where circulating lymphocytes that express this gene present antigens from a pathogen potentially associated with these cellular alterations of the genital epithelium, thus eliciting an immune response that decreases the pathological outcome [64]. Thus, any cellular alteration that the pathogen(s) could have caused is limited, while pups that do not express *Zaca DRB-D* would not adequately implement an acquired response against this hypothesized pathogen, so the alterations caused to epithelial cells would be higher.

A previous study reported a harmful effect of *Zaca DRB-A* for urogenital carcinoma in adult sea lions, where animals that had that gene were four times more likely to develop cancer [22]. In our study, we did not find any protective or harmful effect of specific *Zaca DRB* genes on LSIL or ASCUS. There are several explanations for the apparent discrepancy of results between the two studies. First, we examined the presence of transcribed (i.e. expressed) *DRB* genes, while the abovementioned study assayed the presence of specific genes in genomic DNA, which might not be expressed. Second, we focused on cytological alterations typical of transformation, not on the occurrence of cancerous tumours, which could potentially occur long after initial transformation and at that point be associated with OtHV-1, as has been reported [12,58]. Future studies should aim to include cytologic smear evaluation of free-ranging sea lions, across all age classes, to gain better understanding of epithelial processes in the genital tract.

In conclusion, as recently suggested [1], the California sea lion is likely to become important in the future as a non-traditional model for understanding spontaneous carcinogenesis. Our results constitute exciting evidence that spontaneous or pathogen-induced epithelial transformation is a common occurrence in sea lions, even before sexual maturity, thus furthering our knowledge of cellular transformation in a free-ranging vertebrate population.

## Supplementary Material

Title of ESM 1. Microphotographs of cellular transformation of the genital epithelium of California sea lions from Granito Island.

## Data accessibility

The data used for this study has been stored in Dryad. http://dx.doi.org/10.5061/dryad.7f357.
