# Supplementary material for: Transformation of the genital epithelial tract occurs early in California sea lion development
Source: R Soc Open Sci. 2016 Mar 9;3(3):150419. doi: 10.1098/rsos.150419 (PMC4821252; doi:10.1098/rsos.150419)
Supplement: Title of ESM 1. Microphotographs of cellular transformation of the genital epithelium of California sea lions from Granito Island. [file rsos150419supp1.docx]

Microphotographs of cellular transformation of the genital epithelium of California sea lions from Granito Island


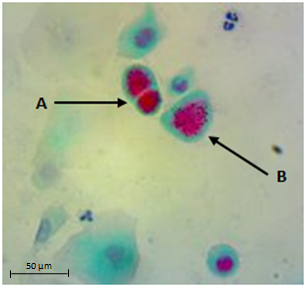

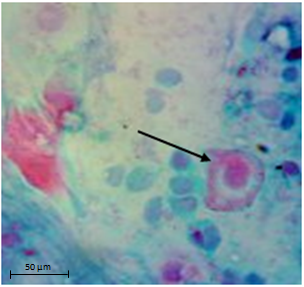


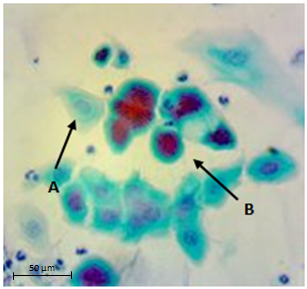

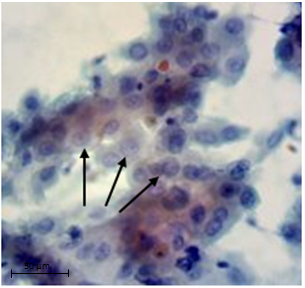


**Top left.** The arrow points to a koilocyte. The clearance around the nucleus is observed. The nucleus is large and there is reinforcement of the cytoplasm relative to the clearance area. **Top right.** A) Binucleated and metaplastic cells. The cell has an irregularly shaped nucleus and altered cytoplasm. The nucleus is hyperchromatic and binucleation is evident. B) Metaplastic cells. **Bottom left.** A). The arrow points to a koilocyte. B) A cluster of metaplastic cells can be observed. **Bottom right.** A large number of reactive cells are observed in the image as indicate dy the arrows. These cells are defined by a zone of perinuclear clearance and prominent nucleoli. All microphotographs were observed at 40x.
